# Difference in prognostic values of maximal standardized uptake value on fluorodeoxyglucose-positron emission tomography and cyclooxygenase-2 expression between lung adenocarcinoma and squamous cell carcinoma

**DOI:** 10.1186/1477-7819-12-343

**Published:** 2014-11-13

**Authors:** Katsuhiko Shimizu, Ai Maeda, Takuro Yukawa, Yuji Nojima, Shinsuke Saisho, Riki Okita, Masao Nakata

**Affiliations:** Department of General Thoracic Surgery, Kawasaki Medical School, 577 Matsushima, Kurashiki, Okayama, 701-0192 Japan

**Keywords:** Non-small cell lung cancer, FDG-PET, SUV, Cox-2

## Abstract

**Background:**

The maximal standardized uptake value (SUVmax) on fluorodeoxyglucose-positron emission tomography (FDG-PET) for primary tumors is correlated with clinicopathological and prognostic factors in patients with non-small cell lung cancer. However, previous investigations have discussed the role of SUVmax without distinguishing among the histological subtypes of lung cancer. Herein, we investigated the correlations among the SUVmax on FDG-PET, clinicopathological or prognostic factors, and the expression of tumor angiogenic biomarkers according to histological subtypes.

**Methods:**

We conducted a retrospective review of data from 52 patients with invasive adenocarcinoma (ADC) and 32 patients with squamous cell carcinoma (SQC) measuring less than 3 cm in diameter. Immunohistochemical staining for cyclooxygenase-2 (Cox-2), Ki-67, and vascular endothelial growth factor, which might influence cancer progression, was performed and the correlations between the expressions of these biomarkers and the SUVmax were evaluated.

**Results:**

Among ADC patients, a statistically significant correlation was observed between the SUVmax and the major clinicopathological factors; among SQC patients, however, no statistically significant association was observed. The disease-free survival (DFS) period of the ADC patients with a high SUVmax was significantly poorer than that of the patients with a low SUVmax, but the DFS of the SQC patients with a high SUVmax was not significantly poorer. In a multivariate analysis, the pathological stage and the SUVmax were independent prognostic factors of the DFS among the ADC patients. Among the SQC patients, however, only Cox-2 expression was an independent prognostic factor of DFS.

**Conclusions:**

Some clear differences in prognostic values of the SUVmax on FDG-PET and Cox-2 expression exist between patients with ADC and those with SQC. Based on these relationships between the SUVmax and clinicopathological or biological factors that influence cancer progression, the importance of the SUVmax appears to be quite different for patients with ADC and those with SQC.

## Background

Fluorodeoxyglucose-positron emission tomography (FDG-PET) has become an important tool for the diagnosis and staging of non-small cell lung cancer (NSCLC) [1]. The maximal standardized uptake value (SUVmax) on FDG-PET is the ratio of the activity in the tissue per unit volume relative to the injected dose according to body weight, and this parameter is widely used because of its simplicity. The SUVmax of primary tumors has been shown to be correlated with the stage, nodal status, histological type, differentiation, and progression of tumors in patients with NSCLC [2–4]. In addition, a high SUVmax has been reported to be a powerful prognostic factor in patients with NSCLC [4–6].

Recently, several studies have reported the existence of a relationship between FDG uptake and the expressions of some molecular biomarkers. Of these, the most famous biomarker related to the SUVmax is glucose transporter 1 [7]. Several other studies have investigated the correlation between FDG uptake and the expressions of biological markers of lung cancer, such as Ki-67, p53, and vascular endothelial growth factor (VEGF) [8–10]. In 2012, we demonstrated that the expression of cyclooxygenase-2 (Cox-2) in tumors was as strongly correlated with a poor clinical outcome as an increase in FDG uptake in lung adenocarcinoma [11].

However, these investigations discussed the role of SUVmax without distinguishing among histological subtypes of lung cancer. Therefore, we investigated the correlation among the expression of selective tumor biomarkers, the SUVmax on FDG-PET, and clinicopathological or prognostic factors according to histological subtypes, specifically adenocarcinoma (ADC) and squamous cell carcinoma (SQC).

## Methods

### Study population

A total of 52 patients with invasive ADC and 32 patients with SQC measuring less than 3 cm in diameter, who had undergone surgical resection with systematic lymph node dissection at Kawasaki Medical School Hospital between 2007 and 2010, were enrolled in this study. We restricted the tumor size to less than 3 cm in diameter because the SUVmax is known to be higher in large tumors [4, 12]. Furthermore, ADC was limited to radiologic ‘invasive’ ADC. Invasive ADC was defined based on the radiologic criteria of a consolidation-to-tumor ratio of greater than 0.50 [13]. None of the patients had received either radiotherapy or chemotherapy prior to the surgery. The histological diagnosis of the tumors was based on the criteria of the World Health Organization, and the TNM stage was determined according to the criteria established in 2009. Written informed consent was obtained from each patient for the study of the excised tissue samples from the surgical specimens. This study was conducted with the approval of the institutional ethics committee of Kawasaki Medical School. (number: 1396, approved on 13 May 13 2013).

### Fluorodeoxyglucose-positron emission tomography

In our institute, all patients with lung cancer had undergone FDG-PET before surgery. However, patients with blood glucose levels of 150 mg/dL or more were excluded from positron emission tomography/computed tomography (PET/CT) acquisition. All PET/CT examinations were performed using a dedicated PET/CT scanner (Discovery ST Elite; GE Healthcare, Tokyo, Japan). PET/CT scanning was performed at 60 minutes after the intravenous injection of 150 to 220 MBq of ^18^FDG (FDGscan, Universal Giken, Nihon Mediphysics, Tokyo, Japan). The regions of interest (ROI) were placed three-dimensionally over the lung cancer nodules. A semi-quantitative analysis of the images was performed by measuring the SUVmax of the lesions. The SUV was calculated based on the following equation:


### Immunohistochemical staining

Immunohistochemical analyses of resected, paraffin-embedded lung cancer tissues were performed. After microtome sectioning (4 μm thickness), the slides were processed for staining using an automated immunostainer (Nexes; Ventana, Tucson, Arizona, United States). The streptavidin-biotin-peroxidase detection technique, using 3,3'-diaminobenzidine as the chromogen, was applied. The primary antibodies were used according to the manufacturer’s instructions (Cox-2: DakoCytomation, CX-294, CA, USA, 1/50 dilution; Ki-67: DakoCytomation, MIB-1, CA, USA, 1/100 dilution; VEGF: Santa Cruz, sc-152, CA, USA, 1:300 dilution). The slides were examined by two investigators who had no knowledge of the clinicopathological data. The expression of each marker protein was examined and evaluated according to a previously reported original protocol. For Cox-2, the slides were scored for the intensity of staining (0 to 3) and the percentages of cells with scores of zero (0%), one (1 to 9%), two (10 to 49%), and three (50 to 100%) were determined. The immunohistochemistry (IHC) score (zero to nine) was defined as the product of the intensity and percentage of the cells. Cox-2 expression was judged as positive when the IHC score was four or more [14]. The labeling index of Ki-67 was measured by determining the percentage of cells with positively stained nuclei. Ki-67 expression was judged as positive when more than 10% of the cancer cell nuclei showed positive staining [15]. VEGF expression was judged as positive when more than 20% of the cancer cell cytoplasm showed positive staining [16].

### Statistical analysis

All the statistical analyses were performed using the SPSS statistical package (version 17.0; SPSS, Chicago, Illinois, United States). Frequencies were compared using the chi-square test for categorical variables, and the Fischer exact test was applied for small samples. Mann-Whitney U tests were performed when comparing continuous variables. Receiver operating characteristic (ROC) curves of the SUVmax for the prediction of recurrence were generated to determine the cutoff value that yielded an optimal sensitivity and specificity. The prognostic evaluation was performed by considering the disease-free survival (DFS) period. DFS was defined as the time until lung cancer recurrence, second cancer occurrence, or non-lung-cancer-related death. The DFS was analyzed using the Kaplan-Meier method. Differences in DFS were assessed using the log-rank test. To assess the potential independent effects on the DFS, we performed multivariate analyses using the Cox proportional hazards model. A *P* value of <0.05 was considered as statistically significant.

## Results

### Clinical characteristics

The characteristics of the patients are summarized in Table 1. A total of 52 patients had invasive ADC, and 32 had SQC. The patients ranged in age from 46 to 83 years (mean: 69.0 years). There were 56 men and 28 women. The median value of the SUVmax of all the tumors was 7.4 ± 4.7 (range: 0 to 18.1). The patient age, sex, and mean SUVmax differed significantly between the histological subtypes.

**Table 1:** **Patient characteristics**

Factor	All cases	ADC	SQC	*P*value
Number	84	52	32	
Age (Mean ± SD)		66.7 ± 8.6	72.7 ± 7.8	0.002
Sex				<0.001
Male	56	24	32	
Female	28	28	0	
Tumor size (Mean ± SD)		22.4 ± 6.9	21.2 ± 6.9	0.423
Pathological stage (%)				0.595
IA	55	36(69)	19(60)	
IB	14	8(15)	6(19)	
IIA + B	6	3(6)	3(9)	
IIIA + B	9	5(10)	4(12)	
SUVmax (Mean ± SD)	7.4 ± 4.7	6.6 ± 5.2	8.8 ± 3.7	0.032

ADC: adenocarcinoma, SQC: squamous cell carcinoma.

### Relationship between the maximal standardized uptake value and clinicopathological findings

Among the ADC patients, a statistically significant correlation was observed between the SUVmax and the degree of tumor differentiation (*P* =0.008), pleural invasion (*P* =0.001), vascular invasion (*P* =0.001), and nodal status (*P* =0.007). In contrast, among the SQC patients, no statistically significant associations were observed between the SUVmax and any of the clinicopathological factors (Table 2).

**Table 2:** **Relationship between the SUVmax and clinicopathological**/**IHC findings**

	ADC	SUVmax		SQC	SUVmax	
Factor	(n =52)	(Mean ± SD)	*P*value	(n =32)	(Mean ± SD)	*P*value
Age			0.778			0.747
<70 years	30	6.4 ± 5.1		11	8.4 ± 4.5	
≥70 years	22	6.9 ± 5.3		21	8.9 ± 3.4	
Sex			0.230			-
Male	24	7.6 ± 5.5		32	8.8 ± 3.7	
Female	28	5.8 ± 4.8		0		
Tumor differentiation			0.008			0.565
Well	30	4.1 ± 3.3		2	12.0 ± 2.1	
Moderate	16	8.5 ± 5.4		21	8.3 ± 3.9	
Poor	6	14.1 ± 2.7		9	9.1 ± 3.6	
Pleural invasion			0.001			0.077
No	38	5.0 ± 4.0		26	8.2 ± 3.7	
Yes	14	11.1 ± 5.4		6	11.2 ± 3.2	
Vascular invasion			0.001			0.602
No	33	4.7 ± 4.0		18	9.1 ± 3.8	
Yes	19	9.9 ± 5.5		14	8.4 ± 3.7	
Nodal status			0.007			0.075
Negative	44	5.6 ± 4.6		26	8.1 ± 3.5	
Positive	8	12.0 ± 4.9		6	11.6 ± 3.7	
Cox-2 expression			<0.001			0.048
Negative	18	2.8 ± 2.4		19	7.7 ± 3.6	
Positive	34	8.7 ± 5.1		13	10.3 ± 3.5	
Ki-67 expression			0.001			0.016
Negative	26	4.3 ± 3.3		15	7.0 ± 4.0	
Positive	26	9.0 ± 5.7		17	10.3 ± 2.9	
VEGF expression			0.004			0.719
Negative	17	3.9 ± 4.1		16	9.0 ± 4.3	
Positive	35	8.0 ± 5.1		16	8.5 ± 3.3	

ADC: adenocarcinoma, Cox-2, cyclooxygenase-2; IHC, immunohistochemical; SQC: squamous cell carcinoma; SUVmax, maximal standardized uptake value; VEGF, vascular endothelial growth factor.

### Relationship between the maximal standardized uptake value and immunohistochemical findings

Among the ADC patients, the Cox-2-positive, Ki-67-positive, and VEGF-positive cases showed a significantly higher SUVmax than the cases that were negative for these markers. On the other hand, among the SQC patients, the Cox-2-positive and Ki-67-positive cases showed a significantly higher SUVmax than the cases that were negative for these markers (Table 2). Using a multiple stepwise regression analysis, Cox-2 expression (*P* =0.007) and Ki-67 expression (*P* =0.028) remained as significant factors that were independently related to the SUVmax in the ADC patients, but only Ki-67 expression (*P* =0.037) remained significant in the SQC patients.

### Prognostic value of maximal standardized uptake value and immunohistochemical findings

**Figure 1:**
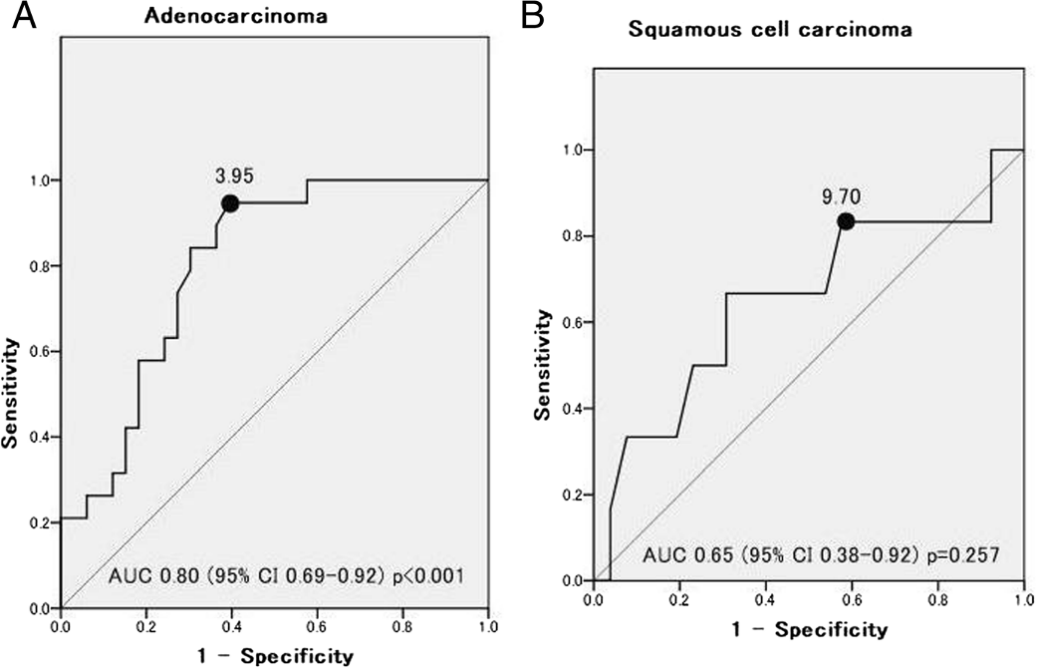
**Receiver**-**operating characteristics (ROC) curve for predicting recurrence. (A)** Adenocarcinoma: AUC 0.80 (95% CI: 0.69 to 0.92), P <0.001. **(B)** Squamous cell carcinoma: AUC 0.65 (95% CI: 0.38 to 0.92), *P* =0.257. AUC, area under the curve; CI, confidence interval.

We used an ROC curve analysis to evaluate whether the SUVmax could predict recurrence (Figure 1). The ROC curves identified an optimal SUVmax cutoff value of 3.95 for predicting recurrence in patients with ADC (area under the curve (AUC) =0.80, *P* <0.001), but not for patients with SQC (AUC =0.65, *P* =0.257). We divided the patient population based on a SUVmax cutoff value of 3.95 for the ADC patients and 9.70 for the SQC patients. Among ADC patients, a high SUVmax was significantly correlated with pleural invasion (*P* =0.027), vascular invasion (*P* =0.042), nodal status (*P* =0.016), Cox-2 expression (*P* =0.001), Ki-67 expression (*P* =0.011), and VEGF expression (*P* =0.003) (Table 3). On the other hand, among the SQC patients, a high SUVmax was not significantly correlated with these factors.

**Table 3:** **Comparison of SUVmax between ADC and SQC**

	ADC: SUVmax			SQC: SUVmax		
Factor	Low	High	*P*value	Low	High	*P*value
Pleural invasion			0.027			0.064
No	19	19		17	9	
Yes	2	12		1	5	
Vascular invasion			0.042			0.735
No	17	16		9	6	
Yes	4	15		9	8	
Nodal status			0.016			0.365
N0	21	23		16	10	
N1 + 2	0	8		2	4	
Cox-2 expression			0.001			0.473
Negative	13	5		12	7	
Positive	8	26		6	7	
Ki-67 expression			0.011			0.087
Negative	15	11		11	4	
Positive	6	20		7	10	
VEGF expression			0.003			0.999
Negative	12	5		9	7	
Positive	9	26		9	7	

ADC: adenocarcinoma, Low/High = SUVmax 3.95. SQC: squamous cell carcinoma, Low/High = SUVmax 9.70. Cox-2, cyclooxygenase-2; SUVmax, maximal standardized uptake value; VEGF, vascular endothelial growth factor.

**Figure 2:**
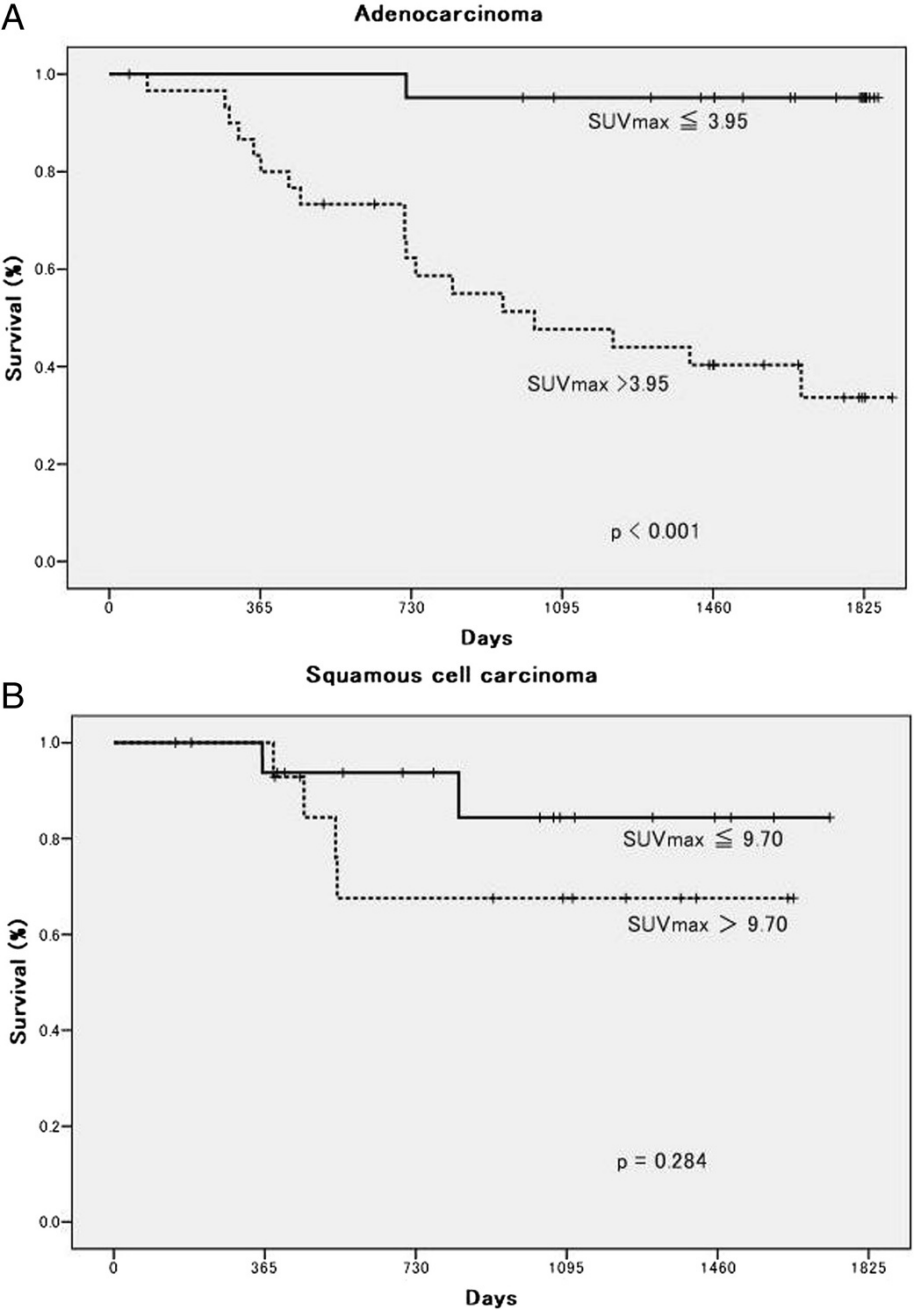
**Kaplan**-**Meier disease**-**free survival curve according to the maximal standardized uptake value (SUVmax).** **(A)** Adenocarcinoma: log-rank *P* <0.001. **(B)** Squamous cell carcinoma: log-rank *P* =0.284.

The DFS of ADC patients with a high SUVmax was significantly poorer than that of patients with a low SUVmax (*P* <0.001, log-rank test; Figure 2A). In contrast, the DFS of SQC patients with a high SUVmax was not significantly poorer than that of patients with a low SUVmax (*P* =0.284, log-rank test; Figure 2B). A univariate analysis of DFS was performed using the variables of pathological stage, SUVmax, Cox-2 expression, Ki-67 expression, and VEGF expression. Among the ADC patients, all the variables were associated with DFS; among the SQC patients, however, only Cox-2 expression was associated with DFS (Table 4). In a multivariate analysis, pathological stage and SUVmax were independent prognostic factors for DFS among the ADC patients, but only Cox-2 expression was an independent prognostic factor for DFS among the SQC patients (Table 4).

**Table 4:** **Results of the univariate and multivariate cox regression analysis of disease**-**free survival**

	Univariate			Multivariate		
	HR	95% CI	*P*value	HR	95% CI	*P*value
A) Adenocarcinoma						
Pathological stage	1.26	1.14-1.10	0.001	1.15	1.02-1.30	0.019
SUVmax	19.42	2.58-146.0	0.001	9.19	1.10-76.54	0.040
Cox-2 expression	4.08	1.18-14.04	0.026	1.57	0.40-6.22	0.521
Ki-67 expression	4.44	1.59-12.38	0.004	1.77	0.54-5.77	0.342
VEGF expression	5.52	1.27-23.94	0.023	1.67	0.36-7.78	0.515
B) Squamous cell carcinoma						
Pathological stage	1.16	0.99-1.36	0.073	1.09	0.88-1.36	0.432
SUVmax	2.46	0.45-13.43	0.300	1.14	0.14-9.40	0.906
Cox-2 expression	9.46	1.10-81.40	0.041	7.36	1.04-72.05	0.048
Ki-67 expression	1.68	0.31-9.20	0.548	1.32	0.23-7.54	0.753
VEGF expression	0.52	0.10-2.84	0.450	0.62	0.89-4.31	0.627

Pathological stage: I versus II + III. CI, confidence interval; Cox-2, cyclooxygenase-2; HR, hazard ratio; SUVmax, maximal standardized uptake value; VEGF, vascular endothelial growth factor.

## Discussion

In this study, we investigated the correlations among the expressions of tumor angiogenic biomarkers, the SUVmax on FDG-PET, and the prognosis according to histological subtypes. As a result, some clear differences in the prognostic value of SUVmax and Cox-2 expression were observed between ADC and SQC. Until now, most publications discussing the correlation between the SUVmax and clinicopathological or prognostic factors have not investigated tumors according to histological subtype. Recently, Tsutani *et al.* reported that the SUVmax of the primary tumor was a powerful prognostic determinant for patients with ADC, but not for those with SQC [17]. The present study provides the evidence showing correlations among the expressions of biomarkers, the SUVmax, and prognosis according to histological subtype. The important findings of this present study were as follows: (i) the SUVmax was correlated with clinicopathological and biological factors in patients with ADC, but not in those with SQC; ii) the SUVmax was a powerful prognostic factor in patients with ADC, but not in those with SQC; (iii) Cox-2 expression was a powerful prognostic factor in patients with SQC, but not in those with ADC. These findings suggest that the SUVmax had no significant value when considering therapeutic strategies for patients with SQC.

The SUVmax of primary lung nodules has been reported to be helpful in distinguishing between malignant and benign tumors, based on the relatively higher values that are seen for malignant tumors [18]. We speculated that tumors showing a higher SUVmax might be more aggressive and might have a higher proliferation potential. We then examined this hypothesis by studying the correlation between the SUVmax and the expressions of some molecular biomarkers, since the overexpression of these markers is known to accelerate tumor progression in NSCLC patients.

Cox-2 expression is highly correlated with tumor angiogenesis and also regulates other angiogenic factors. Some investigators have demonstrated that Cox-2 is constitutively overexpressed in a variety of epithelial malignancies, such as lung, breast, pancreas, colon, esophagus, and head and neck cancers, and Cox-2 overexpression is usually associated with a poor prognosis [19, 20]. To date, some articles have investigated the association between Cox-2 expression and tumor angiogenesis [21]. Recent evidence suggests that Cox-2 plays an important role in tumor-induced angiogenesis through the synthesis of angiogenic prostaglandins, which induce VEGF, and that Cox-2 contributes to neovascularization and may support the vasculature-dependent growth of solid tumors [19]. Our results using resected tissues indicate that the SUVmax of primary tumors might reflect the biological malignant potential, such as tumor angiogenesis. Cancer treatment targeting the control of Cox-2 might become feasible in the future. In 2008, Edelman *et al.* [14] reported that patients with advanced NSCLC tumors with moderate to high Cox-2 expression had a poorer survival outcome than those with a low expression level. Moreover, patients with moderate to high Cox-2 expression had a better tumor response to a Cox-2 inhibitor (celecoxib) in terms of a longer median survival compared with those not receiving celecoxib [14]. On the other hand, in the NVALT-4 study performed in 2011, celecoxib did not improve survival, and Cox-2 expression was not a prognostic biomarker and had no predictive value when celecoxib was added to chemotherapy. However, in a subset analysis, patients with SQC seemed to perform better when treated with celecoxib [22]. Since our results indicate that Cox-2 expression is an independent prognostic factor in SQC, the administration of a Cox-2 inhibitor to patients with SQC seem reasonable.

Immunostaining with the Ki-67 antibody is a widely accepted method for evaluating proliferative activity in a variety of human tumors. Vesselle *et al.* reported the existence of a relationship between the Ki-67 expression index and the degree of FDG uptake in NSCLCs [23]. Also in this study, the expression of Ki-67 was significantly related to the SUVmax in both ADC and SQC. On the other hand, the VEGF family of proteins modulates angiogenesis, which is essential for tumor growth and metastasis. The expression of VEGF has been shown to be associated with tumor angiogenesis, metastasis, and prognosis in several cancers, including NSCLC. To date, two reports have shown a correlation between the expression of VEGF and the SUV on PET-CT [10, 16]. Kaira *et al.* demonstrated a significant correlation between VEGF expression and FDG uptake in NSCLCs [10]. However, our results were not similar to the results of previous report.

A feature of this study was the histological classification of the tumors into ADC and SQC. Recently, clear evidence has suggested that the classification of NSCLC into pathologic subtypes is important for the selection of an appropriate systemic therapy, from the viewpoint of both treatment efficacy and the prevention of toxicity. Pemetrexed yields a much better treatment outcome in patients with ADC than in those with SQC [24]. The use of bevacizumab has been shown to be associated with an increased risk of fatal pulmonary hemorrhage in patients with SQC [25]. Epidermal growth factor receptor mutations is more commonly encountered in ADC [26]. Because of the choice of treatment for SQC, further studies are needed. The present study suggests that a Cox-2 inhibitor might be indicated for the treatment of SQC.

## Conclusions

In conclusion, some clear differences were observed in the prognostic value of Cox-2 expression and the SUVmax on FDG-PET between patients with ADC and those with SQC. Among patients with SQC, Cox-2 expression was a powerful prognostic factor. In patients with ADC, on the other hand, the SUVmax was a potential biomarker of clinical outcome. These findings indicated that the SUVmax of primary tumors might reflect the biological malignant potential in ADC, but the SUVmax had no significant value for determining the therapeutic strategy in patients with SQC. Further study is needed to investigate other factors that might influence the SUVmax on FDG-PET. The small number of patients was a major limitation of the present study.

## Abbreviations

ADC: adenocarcinoma
DFS: disease-free survival
FDG-PET: fluorodeoxyglucose-positron emission tomography
NSCLC: non-small cell lung cancer
ROC: receiver operating characteristic
SQC: squamous cell carcinoma
SUVmax: maximal standardized uptake value.
